# Effects of ethephon on heartwood formation and related physiological indices of *Dalbergia odorifera* T. Chen

**DOI:** 10.3389/fpls.2023.1281877

**Published:** 2024-01-25

**Authors:** Yuan-Jing Zhu, Jia-Wen Li, Hui Meng, Wen-Jie He, Yun Yang, Jian-He Wei

**Affiliations:** ^1^ Institute of Medicinal Plant Development, Chinese Academy of Medical Sciences and Peking Union Medical College, Beijing, China; ^2^ Hainan Provincial Key Laboratory of Resources Conservation and Development of Southern Medicine & Key Laboratory of State Administration of Traditional Chinese Medicine for Agarwood Sustainable Utilization, Hainan Branch of the Institute of Medicinal Plant Development, Chinese Academy of Medical Sciences and Peking Union Medical College, Haikou, China

**Keywords:** *Dalbergia odorifera* T. Chen, heartwood, phytohormone, ethylene, tree physiology

## Abstract

**Introduction:**

*Dalbergia odorifera* T. Chen, known as fragrant rosewood, is a rare and endangered tree species. Studies have shown that plant growth regulators can effectively promote heartwood formation. This study aimed to investigate the effects of ethephon (ETH) on heartwood formation and the influence of ethephon and hydrogen peroxide (H_2_O_2_) on the physiological characteristics in *D. odorifera*.

**Methods:**

*D. odorifera* branches underwent treatment with 2.5% plant growth regulators, including ETH, jasmonic acid (JA), salicylic acid (SA), abscisic acid (ABA), H_2_O_2_, and inhibitors such as ascorbic acid (AsA) to inhibit H_2_O_2_ synthesis, and (S) -trans 2-amino-4 - (2-aminoethoxy) -3-butene (AVG) to inhibit ethylene synthesis. After a 14-day period, we conducted an analysis to evaluate the impact of these plant growth regulators on elongation distance, vessel occlusion percentage, and trans-nerol content. Additionally, the effects of ETH and H_2_O_2_ on endogenous plant hormones, H_2_O_2_ content, soluble protein content, and enzyme activity were investigated within 0-48 h of treatment.

**Results:**

After treatment with ETH for 14 days, the extension distance of the heartwood material was 15 cm, while the trans-nerolol content was 15 times that of the ABA group. ETH and H_2_O_2_ promoted endogenous ethylene synthesis; Ethylene content peaked at 6 and 18 h. The peak ethylene content in the ETH group was 68.07%, 12.89%, and 20.87% higher than the initial value of the H_2_O_2_ group and ddH_2_O group, respectively, and 29.64% higher than that in the AVG group. The soluble protein content and activity of related enzymes were significantly increased following ETH treatment.

**Discussion:**

ETH exhibited the most impact on heartwood formation while not hindering tree growth. This treatment effectively triggered the production of endogenous ethylene in plants and enhanced the activity of essential enzymes involved in heartwood formation. These findings serve as a valuable reference for future investigations into heartwood formation.

## 1 Introduction

Secondary metabolites found in woody medicinal plants, such as terpenoids and flavonoids, possess distinct physicochemical properties and have broad applications in medicine, biomaterials, wood processing, and aromatization (Castel et al., 2005; Aljesri et al., 2014; Yan et al., 2015; Chang and Chang, 2017; Octavia and Nugroho, 2020; Pullaiah et al., 2021; Romruen et al., 2022). Certain resinous woody medicinal plants, under natural conditions without intervention, fail to produce secondary metabolites (Steep, 2003). Instead, the expression of these metabolites is induced by various abiotic stress factors like typhoons and lightning or biotic stress factors, such as ant infestations, insect bites and microbial invasions (Chen et al., 2011). For instance, both argarwood and dragon’s blood are products that form in response to trunk injuries (Chen et al., 2011; Andini et al., 2020). While there are some woody plants that grow naturally under environmental conditions; with age, trees can accumulate secondary metabolites in their trunk, roots, and other parts to form heartwood. However, this process usually takes a long time. Heartwood formation occurs at about 10 years of age in case of *Caesalpinia sappan* L., *Dalbergia odorifera* T. Chen, and *Santalum album* L., and it takes even longer to reach commercial maturity (Liu et al., 2013; Ma et al., 2021; Vij et al., 2023). Due to the immense economic and medicinal value of these woody plants, there is a huge demand in the market. Researchers have focused their efforts on finding effective artificial cultivation methods to expedite the formation of these valuable materials. Studies have shown that biotic stress factors, such as fungal infections (Sun et al., 2015; Cheng et al., 2018), and abiotic stress factors, like drought (Nilsson et al., 2002; Liu et al., 2013; Sun et al., 2020; Li et al., 2021; Cui et al., 2022), can promote the accumulation of secondary metabolites to varying degrees. However, the effectiveness of different treatments varies, and plant growth regulators like ethephon (ETH), jasmonic acid (JA), and herbicides have been extensively studied and have shown notable effectiveness (Radomiljac, 1998; Lin et al., 2000; Li et al., 2021). Furthermore, the effectiveness of plant growth regulators in inducing the accumulation of secondary metabolites varies among tree species. For instance, JA has been found to be more effective in *Aquilaria sinensis* (Lour.) Gilg (Xu et al., 2016), while ETH is reported to be more effective at promoting heartwood formation in *Santalum album* L. and *D. odorifera* (Liu et al., 2013; Cui et al., 2021).

*D. odorifera*, an endemic tree species in China, holds significant value as precious mahogany and traditional Chinese herbal medicine (Tao and Wang, 2010; Zan et al., 2022; Zhu et al., 2023). It has been listed on the International Union for Conservation of Nature (IUCN) Red List by the World Conservation Monitoring Center (WCMC) since 1998 due to the severe depletion of its wild resources (WCMC, 1998). The Chinese government has also promoted it to the second level of national protection. It is of high economic value, easy to survive, and has been widely planted in Guangdong, Guangxi, Yunnan, and other provinces (Liu et al., 2019). However, it is extremely difficult for the tree to become timber. Heartwood formation not only takes over 30 years but also occurs at a very low rate (Ma et al., 2021). It is reported that there are over 2 million artificially planted *D. odorifera* resources in Hainan alone, but the majority of the trees are less than 10-years old, and fewer than 50 are mature trees over 30-years old (Meng et al., 2010). This has triggered a pressing need to identify more effective interventions for promoting heartwood formation. In recent years, researchers have focused on artificial interventions to induce heartwood formation in *D. odorifera*. It has been observed that plant growth regulators and improved forest management practices can effectively stimulate heartwood formation in *D. odorifera*, with ETH being a widely used effective agent. Jia (2014) demonstrated that ETH, 6-benzylaminopurine (6-BA), abscisic acid (ABA), and paraquat all promote an increase in the heartwood area of *D. odorifera*, with ETH producing the largest heartwood area. Cui (2018) noted that ETH treatment induces a nearly uniform color change in *D. odorifera* heartwood and leads to the synthesis of the most comprehensive array of heartwood substances, with the highest content and closest resemblance to natural heartwood, surpassing the effects of JA, salicylic acid (SA), and ABA. Zhou et al. (2014) found that ETH treatment for one year induces *D. odorifera* xylem to produce purplish-red heartwood material, resulting in volatile oil with a Jiangxiang flavor and the highest content of trans-nerolidol, aligning with the primary active ingredient in the Chinese medicine Jiangxiang. Wang et al. (2019) analyzed the effects of four ETH treatment concentrations on the fundamental properties, sugar, starch, histochemistry, and essential oil composition of *D. odorifera* heartwood and found that the 2.5% treatment was most conducive to heartwood formation.

It is evident that various treatments have significant differences with respect to their impact on *D. odorifera* heartwood formation. However, current research primarily focuses on improving the quality of induced heartwood, optimizing induction techniques, and similar aspects, while offering limited insights into the underlying reasons for these differences and the mechanisms at play. Building on our previous research, we have formulated a hypothesis that revolves around the role of endogenous signaling molecules in heartwood response to different treatments. We suggest that phytohormones may serve as pivotal factors, contributing to variations in both the quantity and quality of heartwood formation as a result of their varying responses to external treatments. Therefore, in the present study, we selected four plant growth regulators, namely ETH, ABA, JA and SA, which have been widely reported in the literature and have certain inducing effects, and added the ethylene synthesis inhibitor (S) -trans 2-amino-4 - (2-aminoethoxy) -3-butene (AVG), and compared the differences in their short-term (14 d) effects on the accumulation of heartwood-like substances of *D. odorifera*, based on the physiological and biochemical assays. Meanwhile, based on the early signaling events of ethylene and H_2_O_2_ reported in the literature (Cui et al., 2019), ETH and H_2_O_2_ and their inhibitors were selected in this study to analyze the interaction between endogenous ethylene and H_2_O_2_ in the early period (0-48 h). In addition, we further explored the effects of ETH and its inhibitors on physiological indices, such as endogenous ABA, JA, and SA contents and secondary metabolizing enzyme activities in *D. odorifera*, correlating endogenous key hormones in response to treatments with the formation of secondary metabolites in heartwood in order to use the key hormones as one of the indicators for early prediction of the content of heartwood-like substances, so as to efficiently and rapidly guide the production practice and provide new ideas for the optimization of the formation mechanism and induction technology of the heartwood of *D. odorifera*.

## 2 Materials and methods

### 2.1 Plant materials

The trees, collected from the Haikou Research and Development Center, Hainan Branch, Institute of Medicinal Plants, Chinese Academy of Medical Sciences(Haikou, China), were 5-7-years old unformed heartwood trees in the experimental field. Smooth and straight-sided stems with a diameter of 1–2 cm and long enough (length > 20 cm) were selected.

### 2.2 Treatments and sample pretreatments

**Figure 1:**
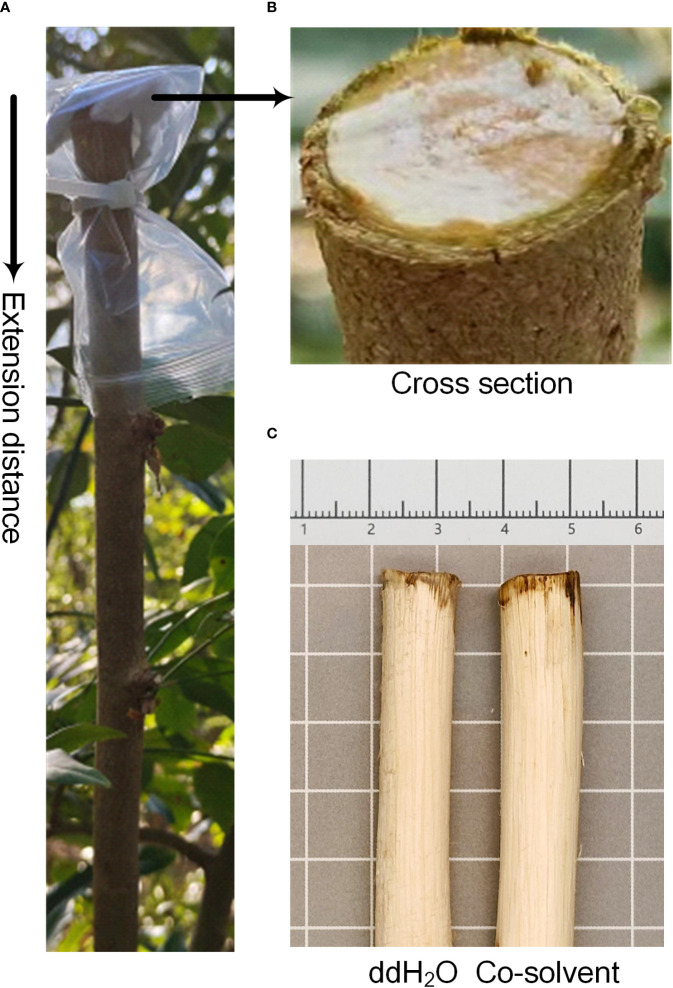
Methods of treatment and pre-experimental results of *D. odorifera* branches. **(A)** Extension distance and arrow illustrate the distance and direction of color change in the branches of *D. odorifera* affected by plant growth regulators. **(B)** Cross section refers to the section of a branch that is still growing on the tree after it has been cut, and also where the skimming cotton is covered. **(C)** Double distilled water (ddH_2_O), co-solvent refers to 95% ethanol.

After the truncation of *D. odorifera* side stems, the cross-sections of branches left on the tree were covered as follows: skimmed cotton saturated with an equal volume of double distilled water (ddH_2_O), 2.5% ETH (analytically pure), 2.5% JA (purity ≥ 95%), 2.5% SA (analytically pure), and 2.5% ABA (HPLC), which the last three reagents required 95% ethanol to help dissolve (400 µL, 380 µL, and 350 µL of ethanol needed to be added per milliliter of solution, respectively). In terms of control selection, we conducted a 14-d preexperiment that showed no difference in the results of treating the branches using equal volumes of ddH_2_O and co-solvent with maximum volume of ethanol to be added (**Figure 1C**), so ddH_2_O was chosen as the control for the next formal experiments. These cotton-covered sections were enclosed within self-sealing bags, securely fastened with ties, and left for 2 h. Each treatment group was replicated three times. After a 14-day period, lateral stems were harvested based on the heartwood extension distance from the branch point (change in color from cross section down). Details are shown in **Figures 1A, B**. A portion of the stem segment was reserved for microscopic observation, while the remaining samples were subjected to 40 °C low-temperature drying and subsequently ground into fine powder using a No.3 sieve. The powdered samples were stored at room temperature (25 °C) for future measurements.

In a separate experiment, following the truncation of *D. odorifera* side stems, the cross-sections were covered with cotton soaked in solutions of the following: ddH_2_O, 2.5% ETH, 0.5 m mol/L AVG (purity ≥ 93%), 1 m mol/L H_2_O_2_ (analytically pure), and 1 m mol/L ascorbic acid (AsA) (analytically pure). These sections were then enclosed in self-sealing bags, securely fastened with ties, and subjected to a 2-h treatment. Subsequently, stem segments of 2 cm in length were collected at intervals of 0, 2, 4, 6, 12, 18, 24, 36, and 48 hours. These segments were immediately placed in liquid nitrogen, ground into a fine powder, and stored in a refrigerator at –80 °C for future measurements.

### 2.3 Statistics of discoloration extension distance and percentage of vessel occlusions (PVO)

After harvesting the branches, the bark was stripped, and the length of the longitudinal discoloration extension was measured. At the end of the extension length measurement, a 2 cm portion of the original section was removed, while the remaining portion was cut into 1 cm stem segments.

CM1950 frozen slicer (Leica, Wetzlar, Germany) was used to create 20–30 μm slices, and each section was observed for ductal obstruction and secondary metabolite formation through a microscope (BCLIPSE80i, Nikon Corporation, Tokyo, Japan). Five views were randomly selected for each section, each view included approximately 15–30 ducts, with a total of approximately 75–150 ducts observed in each section. The percentage of ducts filled with secondary metabolites to the total number of observed ducts was counted (Cui, 2018), i.e., PVO.

### 2.4 Determination of trans-nerolidol content

#### 2.4.1 Preparation of sample solutions

A 0.5 g sample powder was placed in a 20 mL stoppered test tube and mixed with 10 mL methanol (analytically pure). After 1h ultrasonic treatment (SB25-12DTDN, SCIENTZ, Jiangsu, China), filtration treatment was performed. The residue was rinsed twice with 2 mL methanol each time. Subsequently, it was combined with the filtrate to recover methanol under pressure. The residue was rinsed twice with 1 mL ethyl acetate (analytically pure), combined with ethyl acetate to final volume of 2 mL, and the appropriate amount was filtered with 0.45 μm filter membrane. The filtrate was reserved.

#### 2.4.2 Detection conditions

The samples were identified using GC-MS (7890A-5975C, Agilent, New York, USA). The following programme temperature was used: initial temperature of the column 60 °C and held for 2 min, then increased to 90 °C at 6 °C/min and held for l min, 150 °C at 10 °C/min and held for 2 min, 180 °C at 2 °C/min and held for 2 min, 200 at 5 °C/min and held for 2 min, and finally to 250 °C at 2°C/min and held for 2 min; the inlet temperature was 230 °C, the shunt ratio was 2:1. The sample was injected into the sample gate at 230 °C with a shunt ratio of 2:1. The flow rate was 1 ml/min, and the carrier gas was nitrogen (99.999%); the detector was MS, EI ion source at 230 °C, quadrupole temperature at 150 °C, interface temperature at 247 °C, and the solvent was delayed for 3 min; the m/z was 50-500.

#### 2.4.3 Calculation of trans-nerolidol content

The total ion flow map was obtained using GC-MS condition analysis, and the peaks were scanned using mass spectrometry to obtain the mass spectra, which were then searched, analyzed, and identified using the standard mass spectral library. The relative percentage of trans-nerolidol in the volatile oil was calculated using the peak area normalization quantification method.

### 2.5 Determination of relevant physiological indices

Samples collected at 0, 2, 4, 6, 12, 18, 24, 36, and 48 h were subjected to various physiological parameter assays using a UV-1900 ultraviolet-visible spectrophotometer (Shimadzu, Jiangsu, China). Each treatment had three sets of replicates, with each assay requiring 0.1 g of sample powder for a single run. The working solution for the assays was prepared following the instructions provide with the ELISA assay kit (Kejing, Jiangsu, China), and the absorbance (OD) was measured at 450 nm. This allowed us to calculate the content of endogenous ethylene, JA, SA, and ABA. To determine H_2_O_2_ concentration, we used an H_2_O_2_ content kit (Keming, Suzhou,China) and measured the OD at 415 nm. For assessing soluble protein content, we employed the BCA Protein Content Assay Kit (Kejing, Jiangsu,China) and measured the OD at 562nm. To determine the activity of resistance enzymes [Catalase (CAT), peroxidase (POD), and polyphenol oxidase (PPO)], we used kits from Keming (Suzhou, China) and measured the OD at 240 nm, 470nm and 525nm, respectively. Terpene synthase (TPS) (COIBO, Shanghai, China) and chalcone isomerase (CHI) (Keming, Suzhou, China) kits were used to determine the activity of key enzymes involved in the formation of secondary metabolites, and the OD was measured at 450nm and 381nm, respectively.

### 2.6 Data analysis

Data of three replicates in discoloration extension and physiological indices measurements were analyzed in Paired Comparison Plot v3.6 (Origin Lab, Massachusetts, USA), and the mean comparison method is Fisher LSD, the significance level is 0.05.

## 3 Results

### 3.1 Statistics of discoloration extension distance and PVO

**Figure 2:**
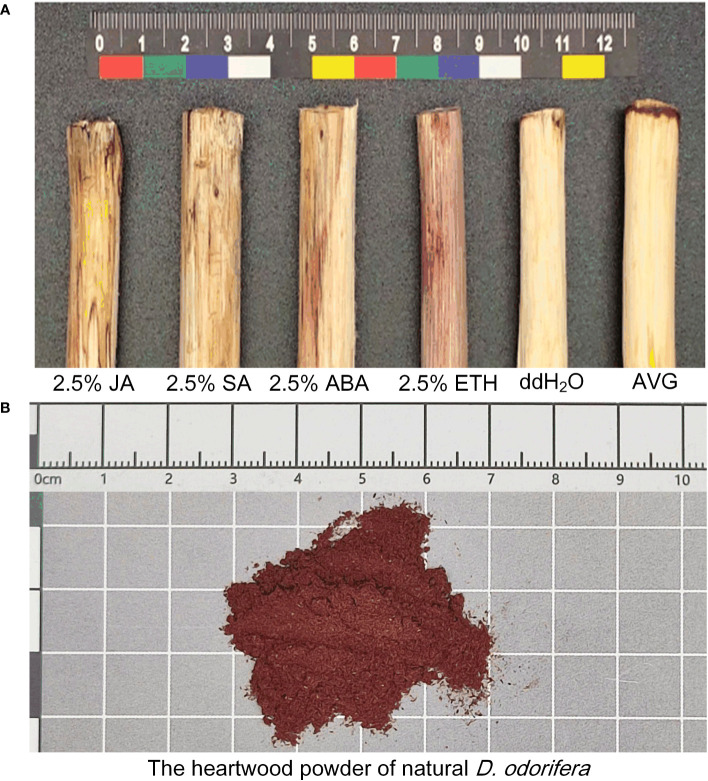
Discoloration of *D. odorifera* branches treated with plant growth regulators for 14 days. **(A)** 2.5% jasmonic acid (JA), 2.5% salicylic acid (SA), 2.5% abscisic acid (ABA), 2.5% ethephon (ETH), double distilled water (ddH_2_O), (S) -trans 2-amino-4 - (2-aminoethoxy) -3-butene (AVG). **(B)** Color of natural *D. odorifera* heartwood powder.

**Figure 3:**
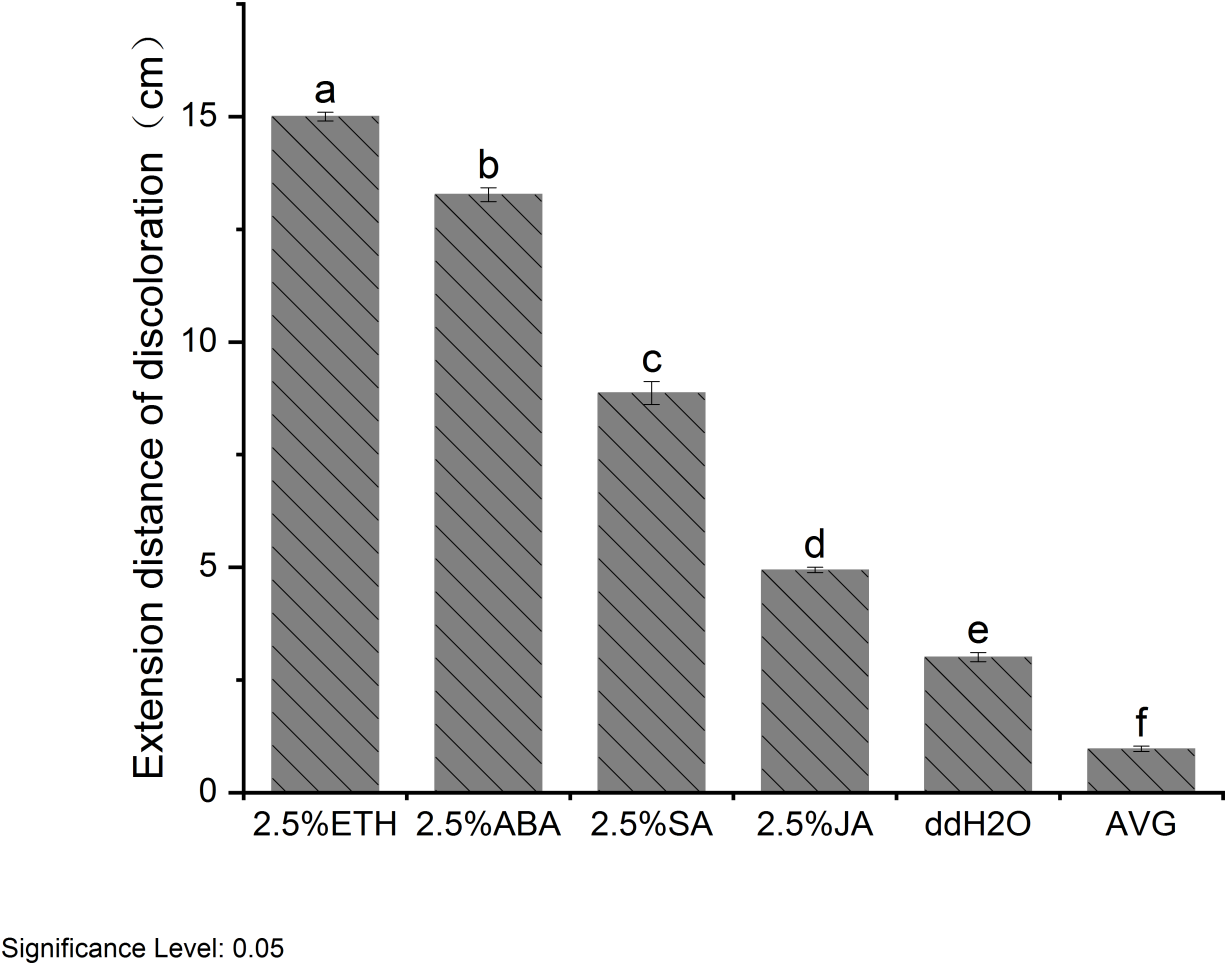
Extension distance of discoloration of *D. odorifera* branches treated with plant growth regulators for 14 days. Values are means ± standard deviation (SD) (n=3), the bars on the top show SD, and different lowercase indicate significant differences among different treatments according to Fisher LSD mean comparison method, respectively (P < 0.05).

When subjected to various plant growth regulator treatments, *D. odorifera* stems exhibited varying degrees of discoloration. Notably, the treatment with ETH closely resembled the natural heartwood color, displaying a rich purple-red hue. This treatment also resulted in the largest discoloration area around the lateral stems, and demonstrated robust branch growth (**Figure 2**). Analysis of extension distance statistics showed that all plant growth regulator treatment groups exhibited greater extension distances compared to the ddH_2_O control group. Among these, the ETH treatment group displayed the longest extension distance, reaching 15 cm, followed by the ABA treatment group with 13.27 cm. In contrast, the JA treatment group had the shortest extension distance of 4.93 cm, while the AVG group showed no discoloration (**Figure 3**).

**Figure 4:**
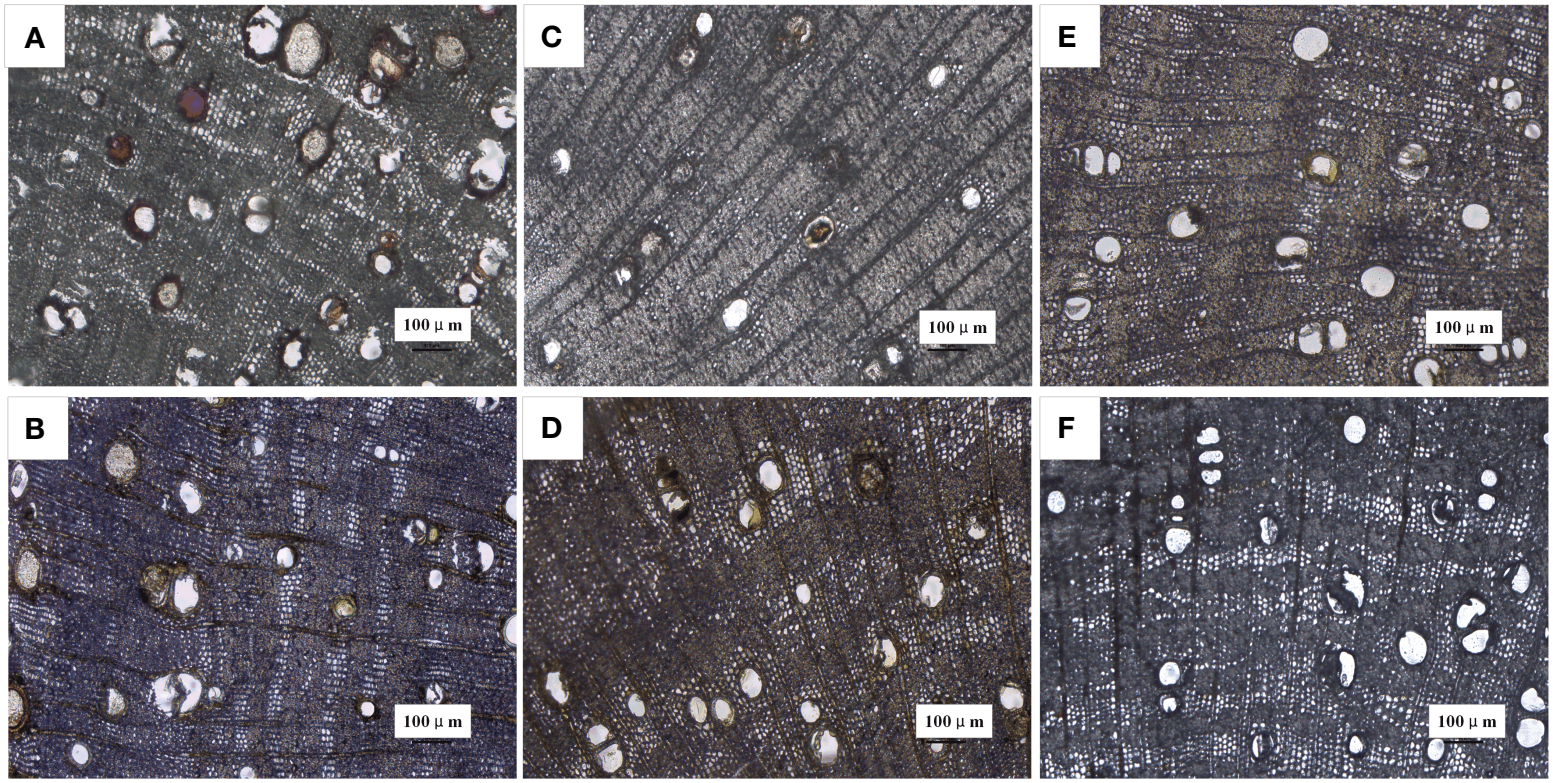
Microscopic observation of vessel occlusions in branches of *D. odorifera* treated with plant growth regulators for 14 days (×10 magnification, 2-3 cm). **(A)** 2.5% ethephon (ETH), **(B)** 2.5% abscisic acid (ABA), **(C)** 2.5% salicylic acid (SA), **(D)** 2.5% jasmonic acid (JA), **(E)** double distilled water (ddH_2_O), **(F)** (S) -trans 2-amino-4 - (2-aminoethoxy) -3-butene (AVG).

During heartwood formation, as thin-walled ray cells undergo cell death, secondary metabolic substances accumulate within the conduits, leading to conduit occlusion (Pallardy and Kozlowski, 2008). The extent of conduit occlusion, referred to as the PVO, is indicative of the quantity of accumulated substances. Therefore, we employed PVO as one of the indicators to compare the effects of different plant growth regulators. PVO measurements were taken from a point below 2 cm, with a decrease observed as the extension distance of discoloration increased. Interestingly, the closer the measurement point was to the tip, the greater the accumulation of filling material and PVO, as depicted in **Table 1**. Notably, the ETH treatment exhibited the most intense purple-red coloration of the conduit-filling material at 2–3 cm (**Figure 4A**). While the ABA (**Figure 4B**), SA (**Figure 4C**), JA (**Figure 4D**), ddH_2_O-treated (**Figure 4E**) groups showed yellow color to varying degrees, the AVG group showed basically no color change (**Figure 4F**).

**Table 1:** Percentage of vessel occlusions in branches of *D. odorifera* treated by plant growth regulators for 14 days.

Slicing depth (cm)	2.5% ETH PVO SD		2.5% ABA PVO SD		2.5%SA PVO SD		2.5% JA PVO SD		ddH_2_O PVO SD	
2–3	69.81%	0.12	53.04%	0.10	55.73%	0.10	31.79%	0.02	9.28%	0.03
4–5	29.86%	0.06	35.63%	0.06	30.63%	0.12	18.32%	0.07	×	×
6–7	26.80%	0.04	20.67%	0.06	16.77%	0.01	×	×	×	×
8–9	26.55%	0.03	11.25%	0.02	3.58%	0.02	×	×	×	×
10–11	13.52%	0.03	9.34%	0.02	×	×	×	×	×	×
12–13	9.56%	0.04	3.33%	0.02	×	×	×	×	×	×
14–15	1.04%	0.02	×	×	×	×	×	×	×	×

### 3.2 Trans-nerolidol content

**Figure 5:**
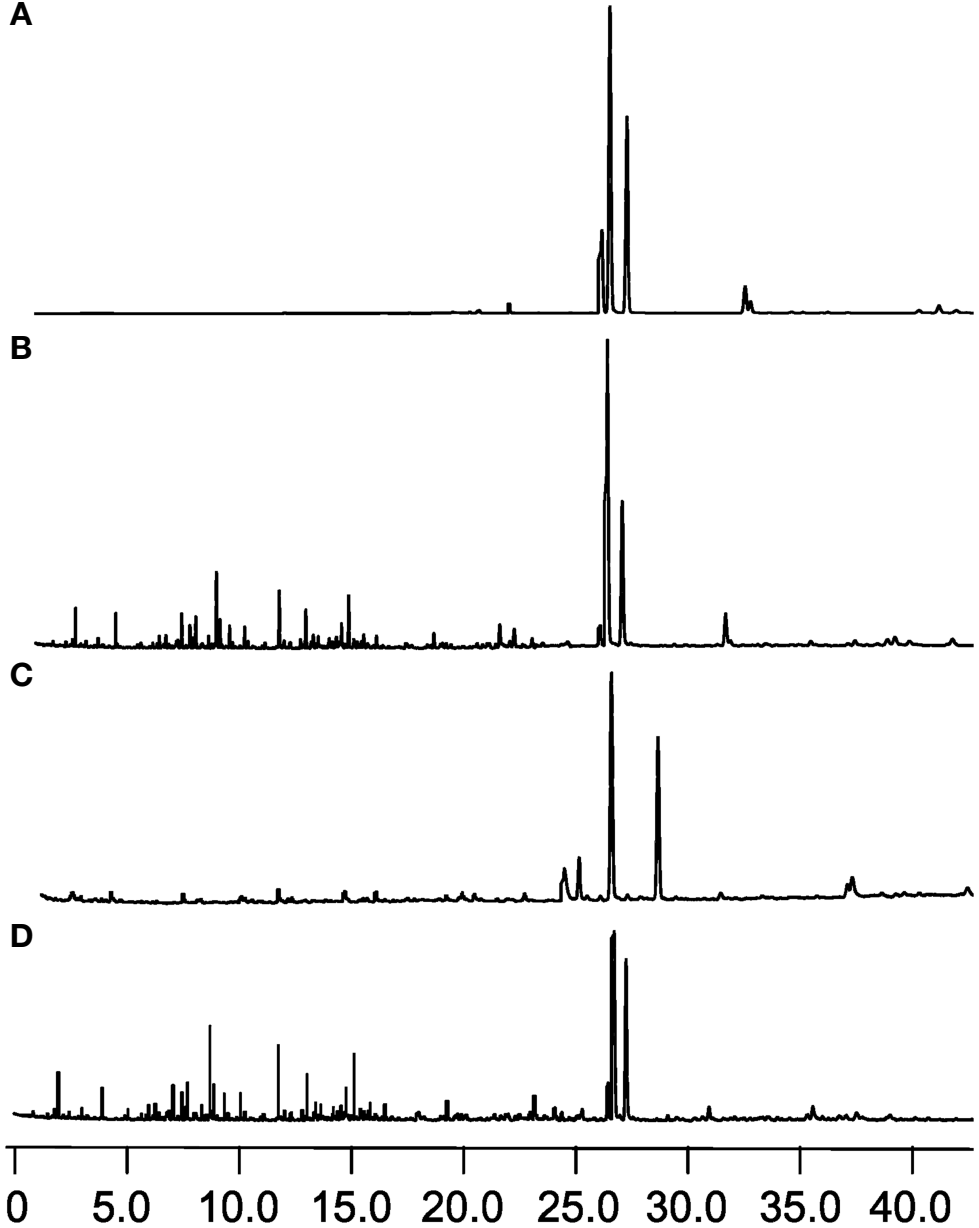
GC-MS total ion chromatograms of *D. odorifera* treated with plant growth regulators **(A)** 2.5% ethephon (ETH), **(B)** 2.5% abscisic acid (ABA), **(C)** double distilled water (ddH_2_O), **(D)** (S) -trans 2-amino-4 - (2-aminoethoxy) -3-butene (AVG).

Trans-nerolidol is the main component of volatile oil in the heartwood of *D. odorifera*. In this study, the trans-nerolidol content was used as one of the evaluation criteria for the effect of different plant growth regulators. in promoting the formation of secondary substances in *D. odorifera*. The total ion flow chromatograms of the volatile oils of different exogenous hormone treatment groups are shown in **Figure 5**, in which the trans-nerolidol content of ETH treatment was 1.7916 mg/g, and the trans-nerolidol content of ABA treatment was 0.1192 mg/g, which was 17.2 and 1.1 times higher than that of the control group of ddH_2_O (0.1041 mg/g), respectively, and the former group was 15 times higher than that of the latter one, whereas the AVG treatment group had the lowest trans-nerolidol content of 0.1022 mg/g; trans-nerolidol was not detected in the lateral stems of the SA and JA treatment groups.

### 3.3 Endogenous hormones and changes in H_2_O_2_

#### 3.3.1 Effect of exogenous ETH treatment on endogenous ethylene and H_2_O_2_ content in the side stems of *D. odorifera*

**Figure 6:**
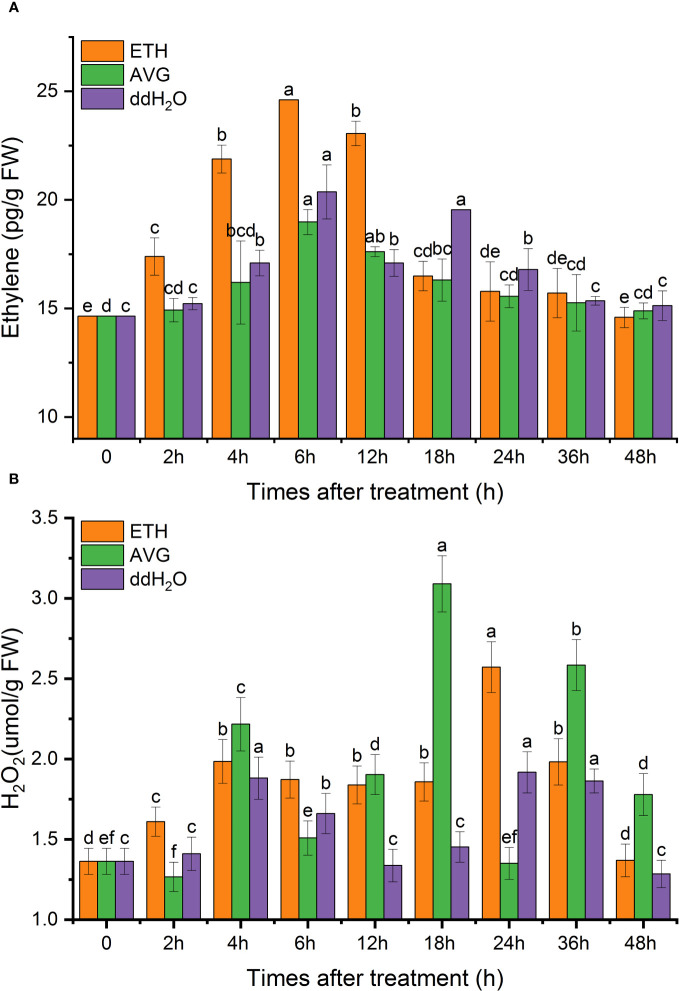
Effects of ETH treatment on the contents of endogenous ethylene **(A)** and H_2_O_2_ **(B)** in *D. odorifera* branches. Values are means ± standard deviation (SD) (n=3), the bars on the top show SD, and different lowercase indicate significant differences among different treatments according to Fisher LSD mean comparison method, respectively (P < 0.05).

The overall endogenous ethylene content in each treatment group first increased, then decreased, and was finally restored to the initial value. In terms of the different treatments, the ETH and AVG groups peaked at 6 h, whereas two peaks appeared at 6 and 18 h in the ddH_2_O group. The peak of the ETH group increased by 68.07% from the initial level, which was 20.87% higher than the highest peak in the ddH_2_O group and 29.64% higher than that in the AVG group, and AVG treatment significantly reduced the synthesis of endogenous ethylene (**Figure 6A**).

After exogenous ETH treatment of *D. odorifera* branches, the H_2_O_2_ content peaked at 4 and 24 h, and the two peaks increased by 45.6% and 88.67%, respectively, compared with the initial value, then gradually decreased to the original level. ddH_2_O group, H_2_O_2_ content change rule, was similar to that of the AVG group, and the H_2_O_2_ content of the AVG group appeared in three peaks at 4, 18, and 36 h. The maximal peak was 2.3 times the initial value, 20.17% higher than that of the ETH group, and the H_2_O_2_ content at 48 h was 1.4 times the initial value, that is AVG did not affect the synthesis of H_2_O_2_ (**Figure 6B**).

#### 3.3.2 Effect of exogenous ETH treatment on other endogenous hormones in the side stems of *D*. *odorifera*

**Figure 7:**
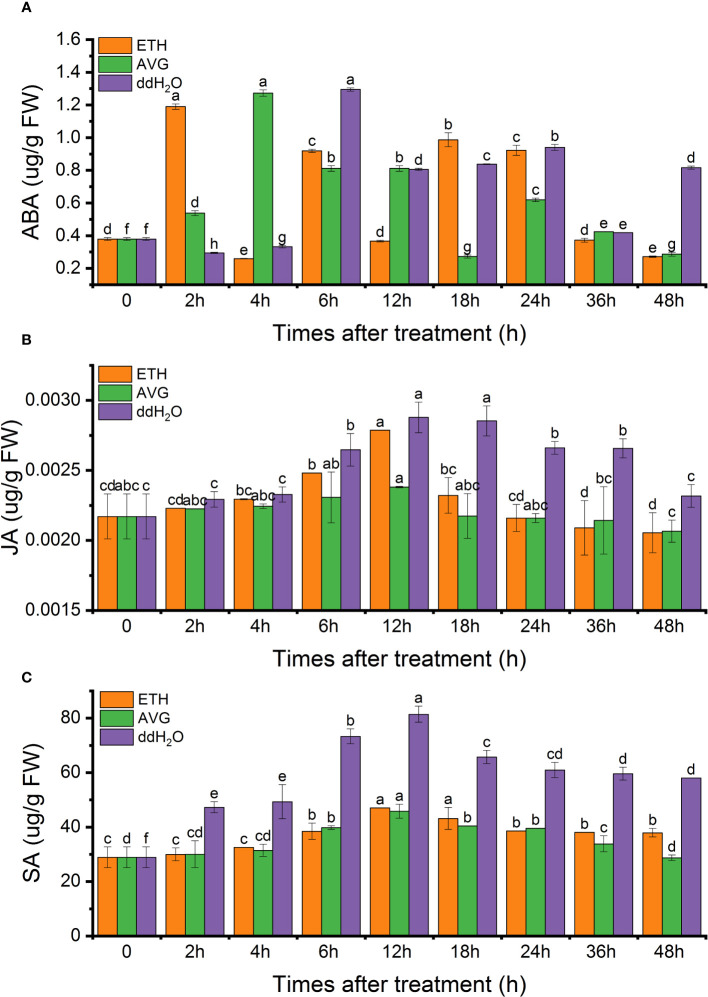
Effects of ETH treatment on the contents of endogenous abscisic acid (ABA) **(A)**, jasmonic acid (JA) **(B)**, salicylic acid (SA) **(C)** in *D. odorifera* branches. Values are means ± standard deviation (SD) (n=3), the bars on the top show SD, and different lowercase indicate significant differences among different treatments according to Fisher LSD mean comparison method, respectively (P < 0.05).

In each treatment group,two or three peaks were observed for endogenous ABA, displaying an overall fluctuating pattern. Notably, the ddH_2_O, AVG, and ETH groups exhibited nearly identical maximum peaks, which were 3.4, 3.36, and 3.14 times higher than the initial values, respectively. In the case of the ETH and AVG groups, the ABA content returned to its initial level after 48 h, while in the ddH_2_O group, it remained at 2.15 times the initial value (**Figure 7A**).

The endogenous JA content exhibited a pattern of initial increase followed by a decrease, reaching its peak at 12 hours in all treatment groups (2.88 ng/g in the ddH_2_O group, 2.79 ng/g in the ETH group, and 2.38 ng/g in the AVG group). Subsequently, the JA content in the ETH and AVG groups gradually decreased to the initial levels, while in the ddH_2_O group, it increased by 6.91% from the initial value after 48 h (**Figure 7B**).

Regarding endogenous SA content, it exhibited a trend of initial increase followed by a decrease, with the peak occurring at 12 h (81.4 μg/g in the ddH_2_O group, 47.06 μg/g in the ETH group, and 45.78 μg/g in the AVG group) across all treatment groups. in the AVG group, the SA content largely returned to its initial level by 48 h. Notably, the endogenous SA content increased by 31.25% compared to the initial value in the ETH group and by 1-fold in the ddH_2_O group (**Figure 7C**).

#### 3.3.3 Effect of exogenous H_2_O_2_ treatment on endogenous ethylene and H_2_O_2_ content in the side stems of *D. odorifera*

**Figure 8:**
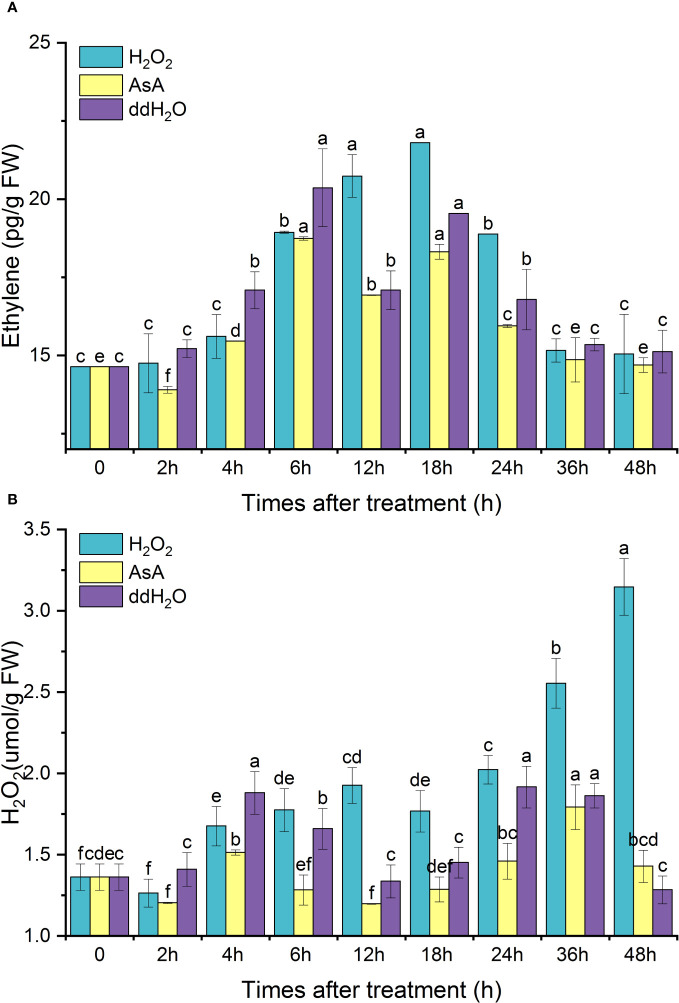
Effects of exogenous H_2_O_2_ treatment on the contents of endogenous ethylene **(A)** and H_2_O_2_ **(B)** in *D. odorifera* branches. Values are means ± standard deviation (SD) (n=3), the bars on the top show SD, and different lowercase indicate significant differences among different treatments according to Fisher LSD mean comparison method, respectively (P < 0.05).

After exogenous H_2_O_2_ treatment, the endogenous ethylene content peaked at 18 h, then gradually decreased to the initial level. The change in the pattern of endogenous ethylene in the AsA group was similar to that in the ddH_2_O group, which was restored to its original level after experiencing two peaks at 6 and 18 h. The peak value of the H_2_O_2_ group at 18 h increased by 48.93% compared with the initial value, 11.58% higher than that of the ddH_2_O group and 19.02% higher than that of the AsA group; AsA inhibited endogenous ethylene synthesis (**Figure 8A**).

After exogenous H_2_O_2_ treatment, the H_2_O_2_ content reached a small peak at 12 h and continued to increase, reaching a maximum peak at 48 h, which was 2.4 times higher than the initial value. ddH_2_O promoted the synthesis of H_2_O_2_, and the overall H_2_O_2_ content was lower in the AsA group (**Figure 8B**).

### 3.4 Soluble protein content

**Figure 9:**
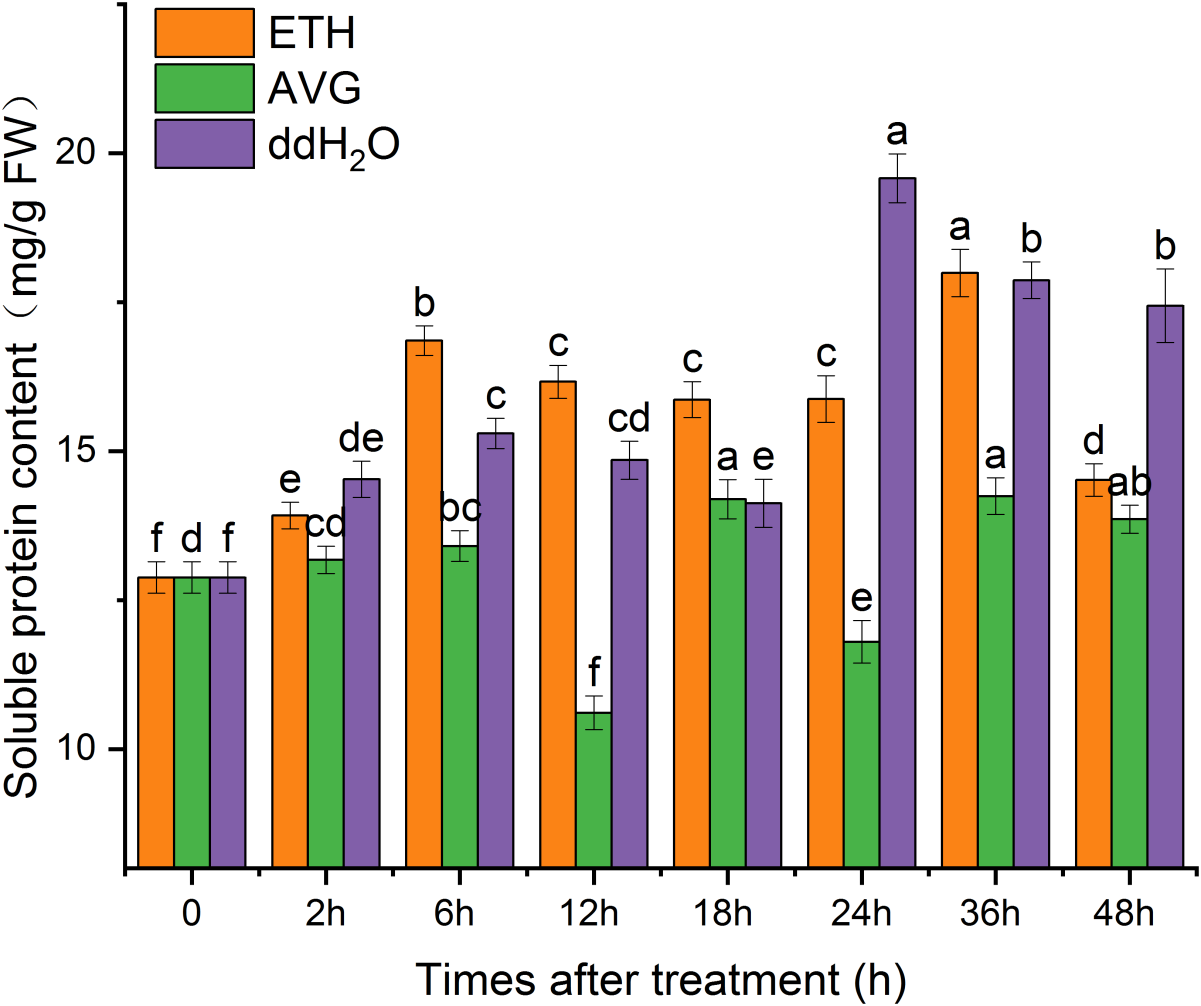
Effects of ETH treatment on soluble protein content in branches of *D. odorifera.* Values are means ± standard deviation (SD) (n=3), the bars on the top show SD, and different lowercase indicate significant differences among different treatments according to Fisher LSD mean comparison method, respectively (P < 0.05).

The majority of soluble proteins found in plants serve as enzymes involved in various metabolic processes, and their concentrations are indicative of the plant’s overall metabolic activity (Wang, 2006). Soluble protein levels in plants not only mirror their overall metabolic activity but also signify their capacity to endure adverse conditions like low temperatures and salt stress (Du and Xiang, 2011; He et al., 2021). The soluble protein content in each treatment group exhibited a fluctuating pattern over the course of 0 to 48 hours; however, they contributed to varying degrees of soluble protein content increase. Following two peaks at 6 and 36 h, the soluble protein content in the ETH group remained at 12.7% higher than the initial value at the 48-hour mark. In contrast, the soluble protein content in the AVG group displayed lower levels and fluctuated around the initial value (**Figure 9**).

### 3.5 Enzyme activity

**Figure 10:**
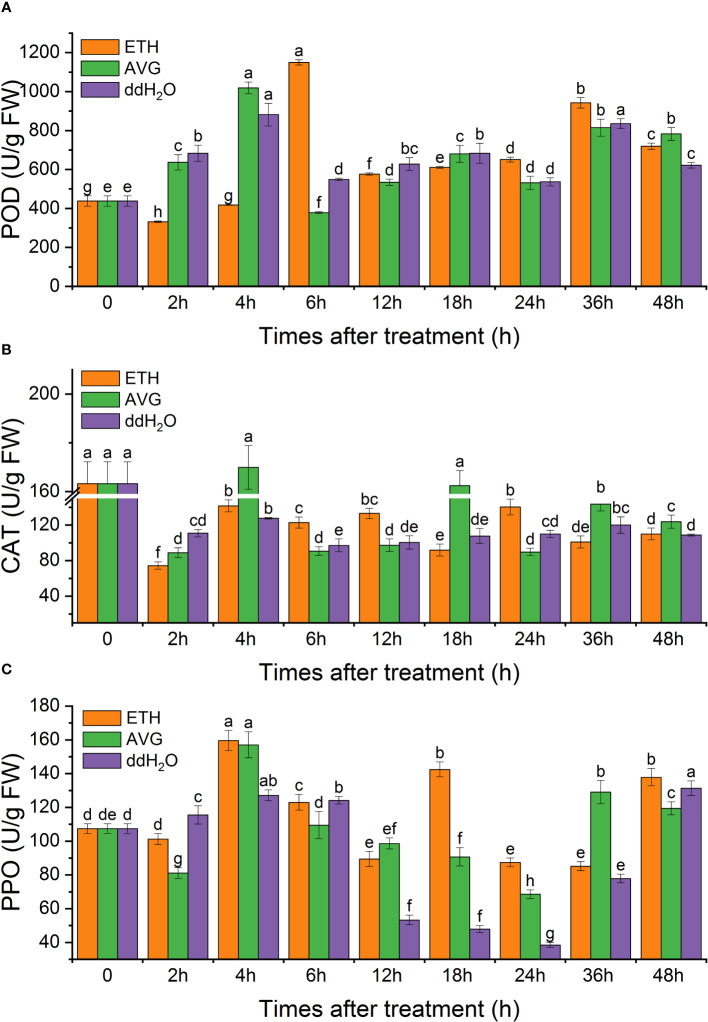
Effects of ETH treatment on the enzymatic activity of peroxidase (POD) **(A)**, catalase (CAT) **(B)**, and polyphenol oxidase (PPO) **(C)** in branches of *D. odorifera.* Values are means ± standard deviation (SD) (n=3), the bars on the top show SD, and different lowercase indicate significant differences among different treatments according to Fisher LSD mean comparison method, respectively (P < 0.05).

The enzyme activities of POD and PPO in each treatment group fluctuated from 0 to 48 h but increased to varying degrees; CAT activity fluctuated above and below the initial level throughout the entire process. The time of the peak activities of the three enzymes coincided with that of the peaks of the contents of ethylene and H_2_O_2_. The activities of POD in each group were 1.42–1.64 times the initial value at 48 h (**Figure 10A**), PPO activity was 1.11–1.28 times (**Figure 10C**), and CAT enzyme activity was lower than the initial level in all groups (**Figure 10B**).

**Figure 11:**
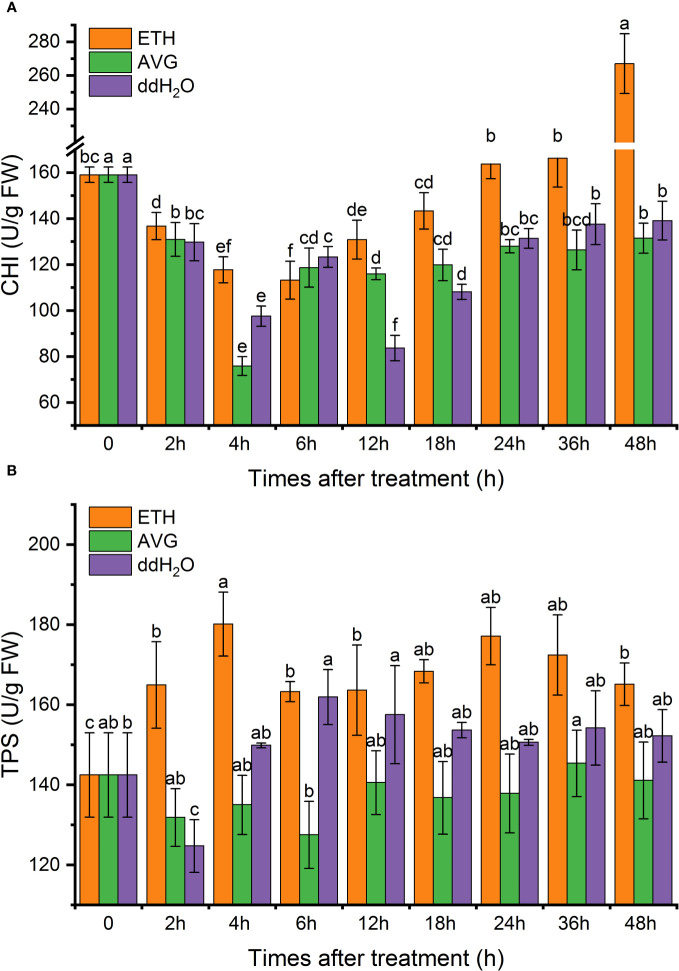
Effects of ETH treatment on chalcone isomerase (CHI) **(A)** and terpene synthase (TPS) **(B)** enzyme activities in branches of *D. odorifera.* Values are means ± standard deviation (SD) (n=3), the bars on the top show SD, and different lowercase indicate significant differences among different treatments according to Fisher LSD mean comparison method, respectively (P < 0.05).

CHI enzyme activity gradually increased after 6 h of exogenous ETH treatment, and the CHI enzyme activity in both ddH_2_O and AVG groups remained around the initial level. At 48 h, the CHI enzyme activity in the ETH group was 67.87% higher than the initial value, 91.94% higher than that in the ddH_2_O group, and 2.03 times higher than that in the AVG group (**Figure 11A**).

The TPS enzyme activity in the ETH group consistently remained 1.1–1.3 times higher than the initial value, which peaked at 4 h. Enzyme activity was 26.41% higher than the initial value, 33.41% higher than that of the AVG group,and 20.18% higher than that of the ddH_2_O group. At 48 h, TPS enzyme activity increased by 15.89% compared with the initial value, 17% higher than that of the AVG group, and 8.48% higher than that of the ddH_2_O group. This suggests that AVG decreased the TPS enzyme activity (**Figure 11B**).

## 4 Discussion

The investigation of abiotic stress modalities, particularly the induction of heartwood formation in *D. odorifera* through plant growth regulators, has primarily focused on long-term trunk treatments, aiming to discern the differences between artificially induced heartwood and naturally occurring heartwood (Jia, 2014; Zhou et al., 2014; Cui, 2018). In contrast, this study adopts a short-term branch treatment approach using cross-section method, evaluating the differential effects of four plant growth regulators. We observed that after a 2.5% ETH treatment sustained healthy growth in *D. odorifera* branches, whereas treatments with 2.5% ABA, 2.5% SA, and 2.5% JA exhibited varying degrees of adverse effects, inhibiting branch growth. Notably, The 2.5% ETH treatment resulted in the longest lateral stem extension, the largest area of color change area, and coloration more closely resembling natural heartwood. Additionally, it led to a relatively high content of the main heartwood component, trans-nerolidol, while AVG treatment showed minimal production of heartwood-related secondary metabolites. Based on these findings, we hypothesize that the plant hormone ethylene may play a pivotal role in *D. odorifera* heartwood formation.

H_2_O_2_ and the phytohormone ethylene function as common signaling molecules in plants, responding to adversity stress with an interactive relationship. Exogenous ETH enhances plant H_2_O_2_ content, consequently promoting endogenous ethylene synthesis, while exogenous H_2_O_2_ can catalyze endogenous ethylene synthesis as well (Li, 2016). Our study revealed that both ETH and H_2_O_2_ treatments triggered an explosive increase in endogenous ethylene content, whereas AVG treatment significantly inhibited endogenous ethylene synthesis. AsA treatment reduced endogenous ethylene content in *D. odorifera* lateral stems, while AVG treatment did not affect the H_2_O_2_ content, implying that H_2_O_2_ likely operates upstream of the ethylene signaling pathway.

Wang et al. (2018) previously demonstrated that treating *D. odorifera* with ETH for two months could regulate the levels of endogenous gibberellin (GA_3_), growth hormone (IAA), ABA, and zeatin riboside (ZR) in response to external disturbances. In this study, we observed dynamic fluctuations in endogenous SA, JA, and ABA contents in ETH-treated side stems of *D. odorifera* over 48 h, although no clear pattern emerged. Given that ddH_2_O treatment induces oxidative damage to the plant to some extent (**Figure 10**) and that SA and JA are vital hormones in abiotic stress responses, SA and JA contents were higher in the ddH_2_O treatment group than in the ETH treatment group. Furthermore, ETH treatment moderately increased the soluble protein content, whereas the AVG treatment group exhibited lower levels of soluble protein content. All treatment groups showed varying degrees of increased antioxidant enzyme activity, indicating dynamic responses to adversity.

*D.odorifera* heartwood primarily contains secondary metabolites such as terpenoids and flavonoids (Liu, 2020; Ninh, 2017). TPS enzymes play a critical role in terpenoid synthesis (Jia et al., 2022), while CHI is a key enzyme in flavonoid synthesis (Yin et al., 2019). Our study revealed that TPS activity significantly increased in the ETH treatment group, remaining consistently high at all time points and peaking at 4 and 24 hours. Conversely, when endogenous ethylene synthesis was inhibited, overall TPS enzyme activity remained low, suggesting ethylene’s involvement in terpenoid compound synthesis. Studies in *Litsea cubeba*, conifers, and Black Walnut also support ethylene’s role in promoting terpene synthesis, especially sesquiterpenes (Phillips et al., 2006; Huang et al., 2013; Wang et al., 2022). Following ETH treatment, CHI enzyme activity gradually increased, peaking at 48 h, whereas the AVG-treated group maintained initial levels, indicating that flavonoid secondary metabolites may not be synthesized at an early stage.

The activity of the vascular cambium layer significantly influences the wood formation rate, with IAA, cytokinins, and ethylene regulating vascular cambium activity to varying degrees (Ye and Zhong, 2015). In Populus, ethylene induces secondary xylem differentiation and promotes increased wood formation (Love et al., 2009). Nevertheless, limited information exists regarding phytohormone involvement in heartwood formation. Existing studies suggest that the transition from sapwood to heartwood is a genetically controlled developmental process, with numerous genes up-regulated during this transition in woody plants (Beritognolo et al., 2002; Jaemo et al., 2004; Huang et al., 2009). While our study links ethylene to heartwood secondary metabolite formation, further investigations involving molecular biology and chemistry experiments are needed to elucidate how *D. odorifera* initiates the ethylene signaling pathway and regulates heartwood compound formation at specific tree ages. This study provides valuable insights into the molecular signaling network of heartwood formation in woody plants.

## 5 Conclusion

In this study, the exogenous application of ETH had profound effects on *D. odorifera* heartwood. It triggered a significant increase in endogenous ethylene content, enhanced overall metabolic activity, and boosted the activities of key enzymes, such as CHI and TPS, involved in the synthesis of secondary metabolites. In contrast, inhibiting endogenous ethylene synthesis markedly reduced these effects. In conclusion, ethylene appears to play a crucial role as a regulatory plant hormone in the formation of *D. odorifera* heartwood compounds. Moreover, our findings suggest that ETH has considerable potential for broader applications in production practices. By expediting the formation of heartwood compounds without hindering normal tree growth, ETH could serve as a valuable tool for accelerating this process. With further refinement and optimization of the inducer formula, ETH has the potential to be widely adopted in practical production. Additionally, concurrently treating both the trunk and lateral branches could enhance overall tree utilization, increase the content and yield of medicinal components, and address the issue of medicinal resource scarcity.

## Data availability statement

The original contributions presented in the study are included in the article/**Supplementary Material**. Further inquiries can be directed to the corresponding authors.

## Author contributions

YJZ: Data curation, Visualization, Writing – original draft. JWL: Methodology, Writing – review & editing. HM: Conceptualization, Funding acquisition, Project administration, Supervision, Writing – review & editing. WJH: Investigation, Writing – original draft. YY: Conceptualization, Supervision, Writing – review & editing. JHW: Conceptualization, Project administration, Supervision, Writing – review & editing.
