# Supplementary material for: Effects of ethephon on heartwood formation and related physiological indices of Dalbergia odorifera T. Chen
Source: Front Plant Sci. 2024 Jan 25;14:1281877. doi: 10.3389/fpls.2023.1281877 (PMC10850394; doi:10.3389/fpls.2023.1281877)
Supplement: Supplementary file 1 [file Table_1.docx]

**Supplementary Table 1.** Extension distance values (mean ± SD, n=3) of discoloration of *D. odorifera* branches treated with plant growth regulator for 14 days.

| Treatments | Extension distance of discoloration (cm) |
| --- | --- |
| 2.5%ETH | 15.00± 0.10^a^ |
| 2.5%ABA | 13.27± 0.15^b^ |
| 2.5%SA | 8.87± 0.25^c^ |
| 2.5%JA | 4.93± 0.06^d^ |
| ddH2O | 3.00± 0.10^e^ |
| AVG | 0.97± 0.06^f^ |

**Supplementary Table 2.** Endogenous ethylene content values (mean ± SD, n=3) in ETH and H_2_O_2_ treatment in *D. odorifera* branches

| Times (h) | Ethylene (pg/g FW) | | | | |
| --- | --- | --- | --- | --- | --- |
|  | ETH | AVG | H_2_O_2_ | AsA | ddH_2_O |
| 0 | 14.64± 0.00^e^ | 14.64± 0.00^d^ | 14.64±0.00^c^ | 14.64±0.00^e^ | 14.64± 0.00^c^ |
| 2 | 17.39± 0.86^c^ | 14.92± 0.54^cd^ | 14.75±0.95^c^ | 13.90±0.11^f^ | 15.22± 0.28^c^ |
| 4 | 21.88± 0.65^b^ | 16.19± 1.91^bcd^ | 15.61±0.70^c^ | 15.46±0.00^d^ | 17.09± 0.59^b^ |
| 6 | 24.61± 0.00^a^ | 18.98± 0.58^a^ | 18.94±0.03^b^ | 18.74±0.05^a^ | 20.36± 1.24^a^ |
| 12 | 23.06± 0.56^b^ | 17.61± 0.23^ab^ | 20.74±0.68^a^ | 16.93±0.01^b^ | 17.09± 0.62^b^ |
| 18 | 16.49± 0.68^cd^ | 16.30± 0.97^bc^ | 21.80±0.00^a^ | 18.32±0.24^a^ | 19.54± 0.00^a^ |
| 24 | 15.78± 1.37^de^ | 15.56± 0.52^cd^ | 18.89±0.00^b^ | 15.95±0.05^c^ | 16.79± 0.97^b^ |
| 36 | 15.71± 1.13^de^ | 15.26± 1.30^cd^ | 15.16±0.37^c^ | 14.86±0.71^e^ | 15.35± 0.20^c^ |
| 48 | 14.58± 0.47^e^ | 14.88± 0.37^cd^ | 15.05± 1.27^c^ | 14.70±0.23^e^ | 15.13± 0.68^c^ |

**Supplementary Table 3.** Endogenous ABA content values (mean ± SD, n=3) in ETH treated *D. odorifera* branches

| Times (h) | ABA (ug/g FW) | | |
| --- | --- | --- | --- |
|  | ETH | AVG | ddH_2_O |
| 0 | 0.38± 0.01^d^ | 0.38± 0.01^f^ | 0.38± 0.01^f^ |
| 2 | 1.19± 0.02^a^ | 0.54± 0.01^d^ | 0.29± 0.00^h^ |
| 4 | 0.26± 0.00^e^ | 1.27± 0.02^a^ | 0.33± 0.01^g^ |
| 6 | 0.92± 0.01^c^ | 0.81± 0.02^b^ | 1.29± 0.01^a^ |
| 12 | 0.37± 0.01^d^ | 0.81± 0.02^b^ | 0.81± 0.01^d^ |
| 18 | 0.99± 0.04^b^ | 0.27± 0.01^g^ | 0.84± 0.00^c^ |
| 24 | 0.92± 0.03^c^ | 0.62± 0.01^c^ | 0.94± 0.02^b^ |
| 36 | 0.37± 0.01^d^ | 0.42± 0.00^e^ | 0.42± 0.00^e^ |
| 48 | 0.27± 0.00^e^ | 0.29± 0.01^g^ | 0.82± 0.01^d^ |

**Supplementary Table 4.** Endogenous JA content values (mean ± SD, n=3) in ETH treated *D. odorifera* branches

| Times (h) | JA (pg/g FW) | | |
| --- | --- | --- | --- |
|  | ETH | AVG | ddH_2_O |
| 0 | 2.17± 0.16^cd^ | 2.17± 0.16^abc^ | 2.17± 0.16^c^ |
| 2 | 2.23± 0.00^cd^ | 2.23± 0.00^abc^ | 2.29± 0.06^c^ |
| 4 | 2.29± 0.00^bc^ | 2.24± 0.02^abc^ | 2.33± 0.05^c^ |
| 6 | 2.48± 0.00^b^ | 2.31± 0.18^ab^ | 2.65± 0.12^b^ |
| 12 | 2.79± 0.00^a^ | 2.38± 0.00^a^ | 2.88± 0.11^a^ |
| 18 | 2.32± 0.13^bc^ | 2.17± 0.16^abc^ | 2.85± 0.11^a^ |
| 24 | 2.16± 0.10^cd^ | 2.16± 0.03^abc^ | 2.66± 0.05^b^ |
| 36 | 2.09± 0.20^d^ | 2.14± 0.24^bc^ | 2.66± 0.07^b^ |
| 48 | 2.05± 0.14^d^ | 2.07± 0.08^c^ | 2.32± 0.08^c^ |

**Supplementary Table 5.** Endogenous SA content values (mean ± SD, n=3) in ETH treated *D. odorifera* branches

| Times (h) | SA (ug/g FW) | | |
| --- | --- | --- | --- |
|  | ETH | AVG | ddH_2_O |
| 0 | 28.88± 3.82^c^ | 28.88± 3.82^d^ | 28.88± 3.82^f^ |
| 2 | 29.95± 2.34^c^ | 30.02± 4.90^cd^ | 47.26± 2.02^e^ |
| 4 | 32.51± 0.00^c^ | 31.43± 2.22^cd^ | 49.28± 6.27^e^ |
| 6 | 38.44± 2.98^b^ | 39.78± 0.65^b^ | 73.25± 2.72^b^ |
| 12 | 47.06± 0.00^a^ | 45.78± 2.58^a^ | 81.40± 2.94^a^ |
| 18 | 43.15± 4.04^a^ | 40.39± 0.00^b^ | 65.71± 2.43^c^ |
| 24 | 38.57± 0.00^b^ | 39.52± 0.00^b^ | 60.93± 2.78^cd^ |
| 36 | 38.03± 0.00^b^ | 33.79± 2.94^c^ | 59.58± 2.37^d^ |
| 48 | 37.90± 1.60^b^ | 28.67± 1.02^d^ | 58.03± 0.00^d^ |

**Supplementary Table 6.** Endogenous H_2_O_2_ content values (mean ± SD, n=3) in ETH and H_2_O_2_ treatment in *D. odorifera* branches

| Times (h) | H_2_O_2_ (umol/g FW) | | | | |
| --- | --- | --- | --- | --- | --- |
|  | ETH | AVG | H_2_O_2_ | AsA | ddH_2_O |
| 0 | 1.36± 0.08^d^ | 1.36± 0.08^ef^ | 1.36± 0.08^f^ | 1.36± 0.08^cde^ | 1.36± 0.08c |
| 2 | 1.61± 0.09^c^ | 1.27± 0.09^f^ | 1.26± 0.09^f^ | 1.20± 0.00^f^ | 1.41± 0.10c |
| 4 | 1.98± 0.14^b^ | 2.22± 0.17^c^ | 1.68± 0.12^e^ | 1.51± 0.02^b^ | 1.88± 0.13a |
| 6 | 1.87± 0.12^b^ | 1.51± 0.11^e^ | 1.78± 0.13^de^ | 1.28± 0.09^ef^ | 1.66± 0.13b |
| 12 | 1.84± 0.12^b^ | 1.90± 0.12^d^ | 1.93± 0.11^cd^ | 1.20± 0.00^f^ | 1.34± 0.10c |
| 18 | 1.86± 0.12^b^ | 3.09± 0.18^a^ | 1.77± 0.13^de^ | 1.29± 0.08^def^ | 1.45± 0.09c |
| 24 | 2.57± 0.16^a^ | 1.35± 0.10^ef^ | 2.02± 0.09^c^ | 1.46± 0.11^bc^ | 1.92± 0.13a |
| 36 | 1.98± 0.14^b^ | 2.58± 0.16^b^ | 2.55± 0.15^b^ | 1.79± 0.14^a^ | 1.86± 0.07a |
| 48 | 1.37± 0.10^d^ | 1.78± 0.13^d^ | 3.15± 0.17^a^ | 1.43± 0.10^bcd^ | 1.29± 0.09c |

**Supplementary Table 7.** Soluble protein content values (mean ± SD, n=3) in ETH treated *D. odorifera* branches

| Times (h) | Soluble protein content（mg/g FW） | | |
| --- | --- | --- | --- |
|  | ETH | AVG | ddH_2_O |
| 0 | 12.88± 0.26^f^ | 12.88± 0.26^d^ | 12.88± 0.26^f^ |
| 2 | 13.92± 0.22^e^ | 13.17± 0.23^cd^ | 14.52± 0.30^de^ |
| 6 | 16.86± 0.25^b^ | 13.41± 0.26^bc^ | 15.30± 0.25^c^ |
| 12 | 16.16± 0.28^c^ | 10.61± 0.28^f^ | 14.85± 0.32^cd^ |
| 18 | 15.86± 0.30^c^ | 14.19± 0.33^a^ | 14.13± 0.40^e^ |
| 24 | 15.88± 0.39^c^ | 11.80± 0.36^e^ | 19.58± 0.41^a^ |
| 36 | 17.99± 0.40^a^ | 14.24±0.31^a^ | 17.87± 0.30^b^ |
| 48 | 14.51± 0.27^d^ | 13.86±0.24^ab^ | 17.44± 0.62^b^ |

**Supplementary Table 8.** POD activity values (mean ± SD, n=3) in ETH treated *D. odorifera* branches

| Times (h) | POD（U/g FW） | | |
| --- | --- | --- | --- |
|  | ETH | AVG | ddH_2_O |
| 0 | 438.27± 26.72^g^ | 438.27± 26.72^e^ | 438.27± 26.72^e^ |
| 2 | 331.11± 4.60^h^ | 636.81± 39.10^c^ | 683.31± 41.22^b^ |
| 4 | 417.24± 1.79^g^ | 1018.82± 30.36^a^ | 881.92± 57.88^a^ |
| 6 | 1149.17± 13.28^a^ | 378.01± 5.09^f^ | 548.94± 6.69^d^ |
| 12 | 576.53± 5.94^f^ | 533.83± 16.19^d^ | 627.94± 33.01^bc^ |
| 18 | 610.58± 4.78^e^ | 680.50± 43.81^c^ | 683.15 ± 51.66^b^ |
| 24 | 650.52± 12.46^d^ | 531.56± 33.94^d^ | 536.66± 20.96^d^ |
| 36 | 942.31± 26.77^b^ | 814.67± 43.34^b^ | 835.33± 25.00^a^ |
| 48 | 719.75± 16.05^c^ | 782.63± 33.11^b^ | 621.23± 14.33^c^ |

**Supplementary Table 9.** CAT activity values (mean ± SD, n=3) in ETH treated *D. odorifera* branches

| Times (h) | CAT（U/g FW） | | |
| --- | --- | --- | --- |
|  | ETH | AVG | ddH_2_O |
| 0 | 163.24± 8.99^a^ | 163.24± 8.99^a^ | 163.24± 8.99^a^ |
| 2 | 74.28± 4.24^f^ | 88.87± 5.46^d^ | 110.96± 4.09^cd^ |
| 4 | 141.63± 6.70^b^ | 169.88± 8.95^a^ | 127.65± 0.92^b^ |
| 6 | 122.74± 6.26^c^ | 90.64± 4.91^d^ | 96.97± 7.21^e^ |
| 12 | 133.12± 5.79^bc^ | 97.26± 6.99^d^ | 100.31± 7.63^de^ |
| 18 | 91.71± 6.69^e^ | 162.44± 6.17^a^ | 107.65± 8.48^de^ |
| 24 | 140.29± 8.87^b^ | 89.69± 4.12^d^ | 109.98± 3.97^cd^ |
| 36 | 100.89± 6.99^de^ | 143.58± 7.72^b^ | 120.04± 9.27^bc^ |
| 48 | 110.00± 6.75^d^ | 123.73± 7.40^c^ | 108.71± 1.06^d^ |

**Supplementary Table 10.** PPO activity values (mean ± SD, n=3) in ETH treated *D. odorifera* branches

| Times (h) | PPO（U/g FW） | | |
| --- | --- | --- | --- |
|  | ETH | AVG | ddH_2_O |
| 0 | 107.35± 2.90^d^ | 107.35± 2.90^de^ | 107.35± 2.90^d^ |
| 2 | 101.18± 3.18^d^ | 81.05± 3.24^g^ | 115.44± 5.39^c^ |
| 4 | 159.51± 6.05^a^ | 157.03± 7.70^a^ | 127.05± 3.22^ab^ |
| 6 | 122.92± 4.70^c^ | 109.43± 8.04^d^ | 124.10± 2.30^b^ |
| 12 | 89.35± 4.41^e^ | 98.49± 3.29^ef^ | 53.23± 2.87^f^ |
| 18 | 142.43± 4.41^b^ | 90.63± 5.45^f^ | 47.87± 2.09^f^ |
| 24 | 87.31± 2.58^e^ | 68.49± 2.54^h^ | 38.47± 1.45^g^ |
| 36 | 85.05± 2.70^e^ | 129.00± 6.84^b^ | 77.81± 2.56^e^ |
| 48 | 137.82± 5.18^b^ | 119.34± 3.85^c^ | 131.34± 4.31^a^ |

**Supplementary Table 11.** CHI and TPS activity values (mean ± SD, n=3) in ETH treated *D. odorifera* branches

| Times (h) | CHI（U/g FW） | | | TPS（U/g FW） | | |
| --- | --- | --- | --- | --- | --- | --- |
|  | ETH | AVG | ddH_2_O | ETH | AVG | ddH_2_O |
| 0 | 159.05±3.35^bc^ | 159.05±3.35^a^ | 159.05±3.35^a^ | 142.52±10.54^c^ | 142.52±10.54^ab^ | 142.52±10.54^b^ |
| 2 | 136.74± 5.87^d^ | 130.97±7.34^b^ | 129.78±8.07^bc^ | 164.99±10.79^b^ | 131.88±7.20^ab^ | 124.77±6.58^c^ |
| 4 | 117.76± 5.64^ef^ | 75.89±4.10^e^ | 97.59±4.41^e^ | 180.16±7.99^a^ | 135.05±7.37^ab^ | 149.90±0.59^ab^ |
| 6 | 113.25± 8.24^f^ | 118.71±8.48^cd^ | 123.33±4.52^c^ | 163.29±2.50^b^ | 127.55±8.38^b^ | 161.96±6.85^a^ |
| 12 | 130.85±8.46^de^ | 115.99±2.57^d^ | 83.69±5.50^f^ | 163.67±11.26^b^ | 140.60±7.97^ab^ | 157.59±12.22^a^ |
| 18 | 143.35±7.91^cd^ | 119.87±6.84^cd^ | 108.13±3.30^d^ | 168.37±2.91^ab^ | 136.83±9.08^ab^ | 153.71±1.91^ab^ |
| 24 | 163.77±6.45^b^ | 127.99±89^bc^ | 131.42±4.24^bc^ | 177.17±7.16^ab^ | 137.92±9.84^ab^ | 150.68±0.74^ab^ |
| 36 | 166.26±12.61^b^ | 126.42±8.64^bcd^ | 137.60±8.85^b^ | 172.45±10.03^ab^ | 145.42±8.29^a^ | 154.24±9.27^ab^ |
| 48 | 267.00±17.81^a^ | 131.51±6.50^b^ | 139.10±8.41^b^ | 165.16±5.31^b^ | 141.16±9.58^ab^ | 152.26±6.54^ab^ |
